# Long-Term Anatomical and Functional Survival of Boston Type 1 Keratoprosthesis in Congenital Aniridia

**DOI:** 10.3389/fmed.2021.749063

**Published:** 2021-09-30

**Authors:** Ariann Dyer, Alix De Faria, Gemma Julio, Juan Álvarez de Toledo, Rafael I. Barraquer, Maria Fideliz de la Paz

**Affiliations:** ^1^Centro de Oftalmología Barraquer, Barcelona, Spain; ^2^Institut Universitari Barraquer, Universitat Autònoma de Barcelona, Barcelona, Spain; ^3^Department of Medicine, Universitat Internacional de Catalunya, Barcelona, Spain

**Keywords:** aniridia, Boston type-1 keratoprosthesis, extrusion, retroprosthetic membrane, aniridia associated keratopathy

## Abstract

**Purpose:** To analyze the long-term anatomical survival, functional survival, and complications of Boston type 1 keratoprosthesis (KPro) in the eyes with congenital aniridia-associated keratopathy (AAK).

**Methods:** A retrospective review of 12 eyes with congenital aniridia that underwent a Boston type 1 KPro surgery was conducted. A Kaplan–Meier analysis was performed. Anatomical and functional success criteria were KPro retention and a best corrected visual acuity (BCVA) ≤1.3 LogMAR (≥0.05 decimal) at the end of a follow-up period. Postoperative complications were recorded.

**Results:** The mean preoperative BCVA was 2.1 ± 0.9 (range: 3.8–1) LogMAR, and glaucoma was a comorbidity in all the cases. Five years after the surgery, the overall retention rate was 10/12 (83.3%), and 50% had functional success. Only three (25%) of the 12 cases did not achieve a BCVA ≤1.3 LogMAR. The cumulative probability of anatomical success was 92, 79, and 79% after 1, 5, and 10 years, respectively. The cumulative probability of functional success was 57 and 46% after 1 and 5 years, respectively. The mean anatomical and functional survival time was 10 ± 1.3 (95% IC = 7.5–12.3 years) and 3.8 ± 0.9 years (95% IC = 1.8–5.8 years), respectively. The most common postoperative complication was retroprosthetic membrane (RPM) formation in 8/16 cases (66%). The mean number of complications per case was 2.4 ± 1.8 (0–6).

**Conclusions:** The Boston type 1 KPro is a viable option for patients with AAK with good anatomical and functional long-term results. Glaucoma is an important preoperative condition that affects functional results. Retroprosthetic membrane formation seems to have a higher incidence in this condition.

## Introduction

Congenital aniridia is a rare condition that affects 1/64,000 to 96,000 live births and has a major impact on vision and, therefore, quality of life (1). Classic aniridia is a panocular phenotypically heterogeneous condition (1, 2) that includes the partial or total absence of an iris, which may be associated with cataracts, foveal and optic nerve hypoplasia, aniridia-associated keratopathy (AAK), and nystagmus (3). It is also associated with mutations in the PAX6 gene. Mutations in other genes contribute to other aniridia-like phenotypes with aniridia-related comorbidities (3–5).

Besides glaucoma, AAK is the most common cause of progressive vision loss, and it can present as early as the first decade of life; however, the median age of diagnosis is 33 years old (4). Aniridia-associated keratopathy is characterized by limbal stem cell deficiency, which causes progressive vascular pannus, corneal conjunctivalization, recurrent epithelial erosions, and subepithelial fibrosis, eventually leading to corneal opacification (2). Because of limbal cell deficiency, penetrating keratoplasty (PK) is usually unsuccessful in the long term, and keratolimbal allografts with or without subsequent PK may be a better alternative; however, aggressive long-term systemic immunosuppression is required (2, 6).

Reported evidence proposes a primary or secondary Boston type 1 keratoprosthesis (KPro) to treat debilitating conditions that carry poor prognoses with PK (2). Traditionally, patients with a KPro have been stratified into three broad categories according to their preoperative diagnoses: (i) recurrent immunologic rejection (including aniridia), (ii) chemical injury, and (iii) autoimmune disease. The first category has a better prognosis when a KPro is implanted. Additionally, a KPro is considered as a treatment option only after several graft failures (7, 8).

The objective of this study was to analyze the long-term anatomical and functional survival and complications of the Boston type 1 KPro in 12 eyes with congenital aniridia. In the last KPro study group meeting (2020), it was suggested that patients with aniridia should be categorized as a separate group rather than be included in the recurrent immunologic rejection category, as previously suggested by other authors (1). This is due to the higher incidence of complications in AAK than most disorders included in this category (1). It is for this reason that this study was conducted.

## Materials and Methods

This original article includes a retrospective review of 12 eyes (10 patients) with congenital aniridia that underwent Boston type 1 KPro surgeries at the Centro de Oftalmología Barraquer, Barcelona, Spain.

The subjects provided written informed consent for the surgery and to participate in clinical research projects at the Centro de Oftalmología Barraquer, Barcelona, Spain. Institutional review board approval was obtained for the retrospective review of the clinical records of the patients, and this study adhered to the tenets of the Declaration of Helsinki.

The surgical technique used for the implantation of the Boston type 1 KPro has been described widely (9). In summary, an 8.5- to 9-mm corneal donor button was trephined, and a 3-mm opening was created at its center with a punch. The optical stem of the KPro was placed through the central hole, the back plate was located in place, and then a titanium locking ring was fixed into a groove in the optical stem. Finally, the cornea was sutured as in a standard PK. If an eye was pseudophakic, the intraocular lens (IOL) was left in place; if it was phakic, the lens was removed, making the eye aphakic.

Postoperative treatment includes the use of a permanent Proclear^®^ (CooperVision Inc, Scottsville, NY, United States) soft contact lens that is 16-mm diameter, and an 8.8-mm base curve placed at the end of the surgery, along with wide-spectrum topical antibiotics (ciprofloxacin 3 mg/ml TID for 1 week and vancomycin 14 mg/ml BID to be continued for life), prednisolone acetate 1% (three to five times per day) with slow taper for 1 month, and anti-glaucomatous treatment as deemed necessary. A fornix rinse with povidone-iodine was performed every month in all the patients after the 1st month of the surgery.

A retrospective review of the medical records of all patients with congenital aniridia who underwent Boston type 1 KPro implantations from December 2010 to October 2019 was conducted at the Centro de Oftalmología Barraquer. Three experienced surgeons (AT, RIB, and MdP) implanted the Boston type 1 KPro in all the studied eyes.

Gender, age at surgery, visual acuity (VA), aniridia-related comorbidities, anatomical retention period, follow-up time, and postoperative complications were recorded and analyzed. The pre- and postoperative best corrected visual acuity (BCVA) of all the patients were measured and recorded on a decimal scale and then converted into a LogMAR scale for statistical analysis.

The main outcomes were graft retention and the BCVA. Secondary outcomes were postoperative complications.

A Kaplan–Meier survival analysis was performed. Anatomic success was defined as prosthesis retention during the follow-up period. Extrusion was considered as the exposure of the back plate due to corneal melting that needs surgical resolution such as a lamellar patch or KPro exchange.

Functional success was defined as BCVA ≤1.3 LogMAR (≥0.05 decimal) at the end of the follow-up period. The mean time of functional success was the estimated mean time with a maintained BCVA ≤1.3 LogMAR (≥0.05 decimal).

The follow-up period represents the time between the KPro surgery and the last recorded appointment with no signs of extrusion, or the interval of time between the KPro surgery and the first extrusion, which is when it was diagnosed. The follow-up period for the functional survival analysis was the time with a maintained BCVA ≤1.3 LogMAR.

## Results

### Demographic Data

The 12 eyes of 10 patients were studied. There were three (30%) male and seven (70%) female patients. Nine (90%) of the patients were Caucasian, and one (10%) was African. The mean age at surgery was 37.1 years (range: 5–63 years). The mean follow-up time was 58.7 ± 41.4 months (range: 9–142 months).

Table 1 shows a list of individual demographic and clinical preoperative characteristics. The Boston type 1 Kpro was a primary indication in seven eyes (58%), and the secondary Boston type 1 Kpro was performed in five eyes (42%), which had undergone previous PKs that, in some cases, had multiple failed attempts. Five of the eyes (42%) had previously undergone ocular surface stem-cell treatments (limbal allografts) without success.

**Table 1:** Demographics and clinical preoperative features in each case.

**Eye code**	**Age range**	**Gender**	**Preoperative BCVA (LogMar)**	**Previous surgeries**	**Comorbid conditions**	**Additional procedure**
1	35	F	2.1	LA, PK, CE+IOL, S	N, OSD,G, FH,CC	
2	37	F	1.3	CE+IOL,S	N, OSD,G, FH,CC	IOLR
3	72	M	3.2	CE	N, OSD, G,CC	
4	5	F	1.3	CE,S	OSD,G, FH,CC	V
5	6	F	2.3	CE,S	OSD,G, FH,CC	V
6	35	F	1.7	CE+IOL,GS	OSD,G	
7	63	F	1	LA, PK,CE+IOL,GS, SIOL	N, OSD,G, FH	
8	13	M	3.8	PK, CE+IOL,GS	N, OSD,G, CC	
9	33	F	3.2	LA, CE+IOL, GS	N, OSD,G, CC	
10	51	M	2.1	PK, CE+IOL	N, OSD,G, FH,CC	
11	38	F	1.6	LA, CE+IOL, GS, E	N, OSD,G, CC	
12	57	F	1.5	LA, PK,CE+IOL	OSD,G, CC	

*CC, congenital cataract; CE + intraocular lens (IOL), cataract extraction with IOL; CE, cataract extraction; E, epitheliectomy; F, female; FH, foveal hypoplasia; G, glaucoma; GS, glaucoma surgery; IOLR, IOL removal; M, male; N, nystagmus; LA, limbal allograft; OSD, ocular surface disease; PK, penetrating keratoplasty; RD, retinal detachment; S, strabismus; SIOL, suture of the IOL; V, vitrectomy*.

Most of the patients had poor preoperative visual acuities. The mean preoperative BCVA was 2.1 ± 0.9 LogMAR (range: 3.8–1 LogMAR). Aniridia-related comorbidities, such as nystagmus, were present in eight of the 12 cases (67%) and foveal hypoplasia was in six of the 12 cases (50%). All of the cases had both ocular surface diseases and glaucoma. Previously operated congenital cataracts were reported in 10 of the 12 cases (83%), and none of our patients suffered retinal detachments before the Boston type 1 KPro surgery.

### Anatomical Results

**Figure 1:**
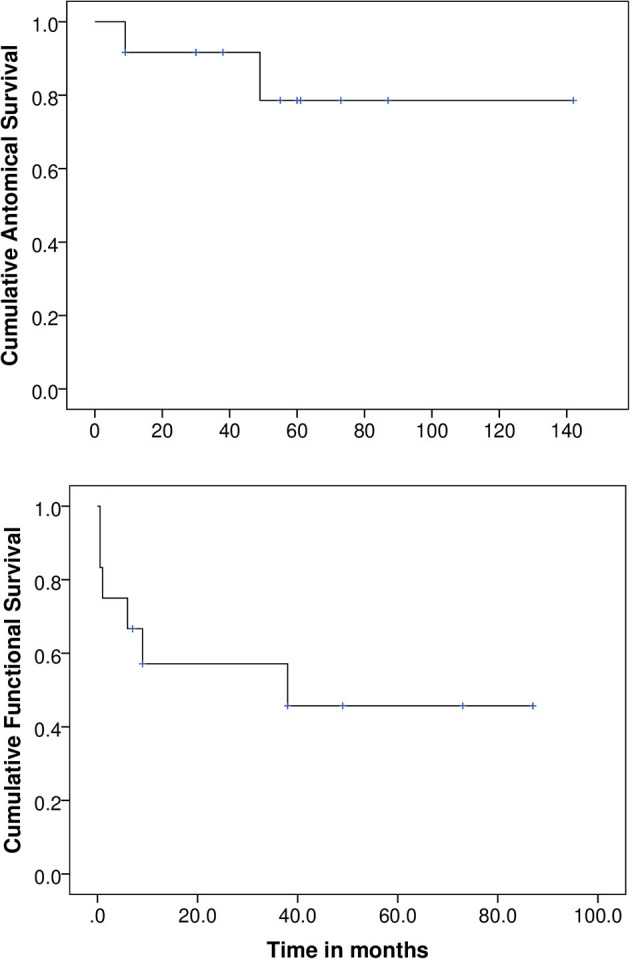
Anatomical and functional survival analysis.

The overall retention rate of the Boston type 1 KPro 5 years after the surgery was 83.3% (10/12). The mean follow-up for the anatomical survival analysis was 53.6 ± 36.6 months (range: 9–142 months). The cumulative success rate by the Kaplan–Meier analysis was 92, 79, and 79% after 1, 5, and 10 years, respectively. Figure 1 illustrates these results. The mean anatomical survival time was 119 ± 15 months (95% CI = 90–148 months). Failures appeared in both eyes of the same patient. One of the failures underwent the first extrusion 9 months after the surgery. After that, several tectonic surgeries had to be performed because of continuous extrusions (see Table 2 for individual details). The second eye had its first extrusion after 49 months of follow-up and required a transmucosal Boston type 1 KPro procedure, as described by Camacho et al. (10), because of repeated extrusions that were not solved with tectonic grafts. The functional success was achieved in both cases before the first extrusion and both eyes maintained BCVA <1.3 LogMAR (≥0.05 decimal) during 15 and 55 months respectively after secondary rescue procedures, although visual acuity was progressively decreasing in one case.

**Table 2:** Individual data of postoperative complications and procedures.

**Eye code**	**Postoperative complication**	**PostKPro ocular procedures**
1	GEm, RPMm	YM, YC, LTK, GDD, KProE
2	GEm, RPMm	YM, LTK, GDD,
3	SU, UIOP	TT
4	RPMm	YM
5	RPM, E	PPV
6	-	-
7	RPMm, RD	YM, PPV
8	RD	PPV
9	MK, RPM	YM
10	-	YC
11	RPM, RD	YM, PPV
12	SU, RPM, RD	PPV

*GE, graft extrusion; MK, microbial keratitis; SU, sterile ulceration; WD, wound dehiscence; RPM, retroprosthetic membrane; RD, retinal detachment, UIOP, uncontrolled IOP; E, endophthalmitis; YM, yttrium-aluminum-garnet (YAG) membranotomy; YC, YAG capsulotomy; LTK, lamellar techtonic keratoplasty; GDD, glaucoma drainage devices; KProE, Boston type 1 keratoprosthesis (KPro) exchange; TT, temporal tarsorrhaphy; PPV, pars plana vitrectomy; m, multiple*.

### Functional Results

The overall rate of functional success 5 years after surgery was 50% (6/12). The eyes maintained a BCVA ≤1.3 LogMAR (≥0.05 decimal) throughout the entire follow-up period and improved their final BCVA with a mean difference of 0.7 ± 0.5 LogMAR (range:0.2–1.7 LogMAR), compared with their preoperative BCVA.

The mean follow-up for the functional survival analysis was 26.5 ± 30.2 months (range:0.5–87 months). Figure 1 illustrates the cumulative probability of success by the Kaplan–Meier analysis, which was 57 and 46% at 1 and 5 years, respectively. The mean functional survival time was 46 months (95% CI: 22–69 months).

Of the six functional failures, three eyes did achieve a BCVA ≤1.3 LogMAR after the KPro surgery during a period of 6, 9, and 38 months, with a maximum improvement of 2.15, 0.6, and 0.25 LogMAR, respectively, compared with the preoperative BCVA. However, other complications, such as uncontrolled intraocular pressure (IOP) (in one case) and retinal detachments with macular involvement (in two cases), compromised the VA, resulting in functional failure.

**Figure 2:**
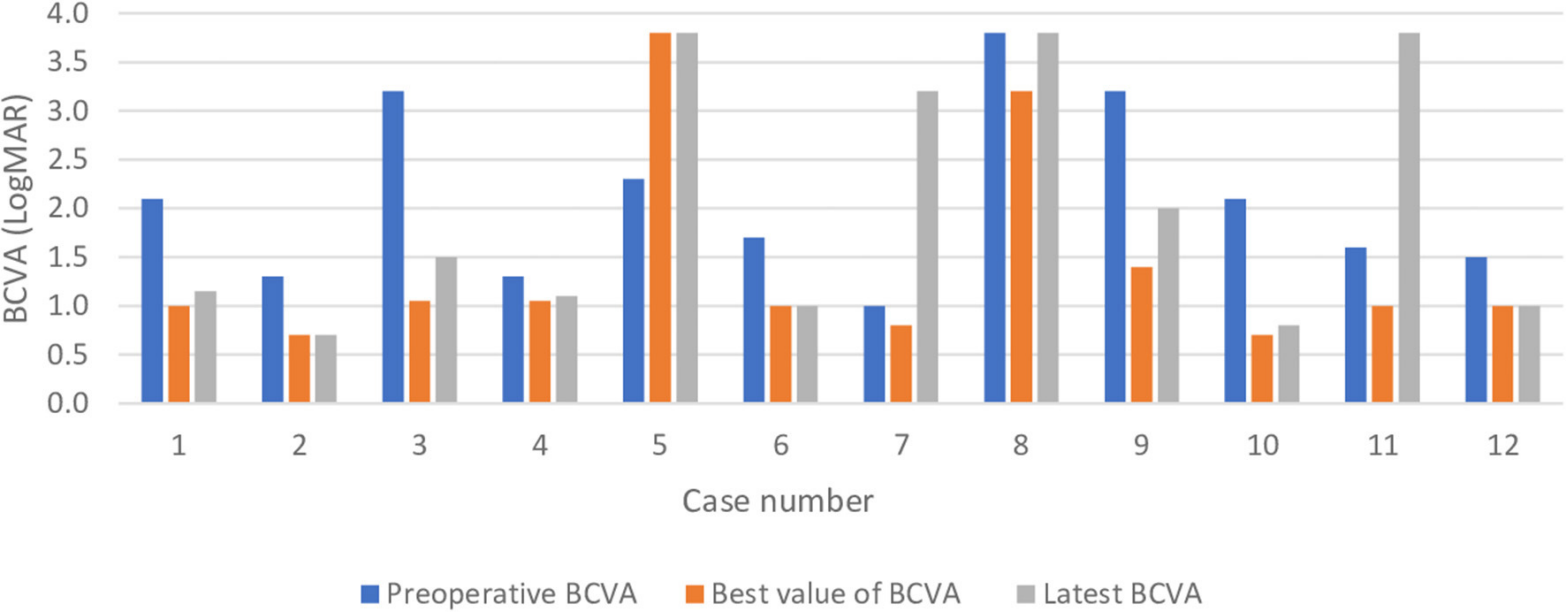
Best corrected visual acuity changes in the sample.

Only three cases (25%) never achieved a BCVA ≤ 1.3 LogMAR, even though two of these eyes did improve their VA (Figure 2 illustrates the BCVA changes in each case studied). Out of these three eyes, two started with very low VA (hand movement or light perception) due to aniridia-related comorbidities. The other eye suffered postoperative endophthalmitis that led to phthisis bulbi.

### Postoperative Complications

The most common postoperative complications encountered were prosthesis extrusion (2/12−17%-, explained in anatomical results) and retroprosthetic membrane (RPM) formation (8/12−66%-). In five of the cases (40%), the complication occurred before 2 years, and in three (25%) after 2 years. The recurrence of RPM was the most frequent complication in each case, occurring up to three times in the same eye. Each case that required it was resolved by membranotomy with the yttrium-aluminum-garnet (YAG) laser (Lumenis Selecta® Duet™, Salt Lake City, UT, United States). Other complications found were retinal detachment (33%), microbial keratitis (8%), sterile ulceration (8%), and endophthalmitis (8%). The mean number of complications per case was 2.4 ± 1.8 (range: 0–6).

Table 2 illustrates postoperative individual complications and procedures after Boston type 1 KPro in aniridia.

## Discussion

Aniridia-associated keratopathy is a major threat to vision in patients with aniridia, and it occurs in up to 90% of cases (1, 2, 11). This is the result of limbal stem cell deficiency, which leads to progressive corneal scarring and eventual blindness (1). This condition is very challenging to manage, and conventional PK has been proven to be ineffective because it fails to address the underlying cause; (2) PK with keratolimbal allografts requires serious systemic inmunosupression because of its higher than usual rate of immunological rejection (11). A keratoprosthetic implantation has become a treatment option in the past decade for severe conditions that have low probabilities of success, and the Boston type 1 KPro is the one that is most commonly used, even as a primary indication (1, 2).

All the cases in this study had poor preoperative visual acuities (BCVA ≥ 1 LogMAR, 0.09 decimal scale), as seen in previous studies on patients with aniridia (1, 2, 6, 11). We found that the rate of preoperative glaucoma (100%) was slightly higher than that described in publications (around 80%) (1, 2). The long follow-up period of this cohort, compared with published studies evaluating the clinical outcomes of patients with the congenital aniridia spectrum after implantation with the Boston type 1 KPro, strengthens the reliability of our results.

The KPro overall retention rate of 83.3% is lower than that found by Rixen et al., Akpek et al., and Bakhtiari et al. (100%), but is similar to that found by Hassanaly et al. (77%) and Shah et al. (87%) (1, 2, 5, 6, 11). This could possibly be attributed to the longer follow-up period, with one of the extrusions occurring after 49 months (4 years). This is longer than the median follow-up time published by some authors (2, 6, 11). Another reason could be our strict definition of extrusion; no other authors clarified what they considered an extrusion and, in one case, anatomical success was considered even after the KPro had to be changed (5). The probability of retention of the Boston type 1 KPro in our study population was 92, 79, and 79% after 1, 5, and 10 years, respectively.

The failures appeared to be due to corneal melting in both eyes of the same patient (9 months and 49 months after surgery). This could be due to an ocular surface/immunological personal predisposition. Several tectonic surgeries had to be performed in the first extruded KPro, and the second one required a transmucosal Boston type 1 KPro exchange after many failed tectonic grafts. This surgical technique may be considered an alternative in patients with ocular surface/immunological diseases (10). Other treatment options include a Boston type 2 KPro, osteo-odonto keratoprosthesis (OOKP), and osteo-keratoprosthesis (OKP); however, these are usually indicated in eyes with advanced-stage ocular surface diseases (12, 13). There are limited outcome data on the Boston type 2 KPro in the literature (12), and fewer regarding type-2 implants or OOKP in patients with aniridia (12, 13). This could be because patients with aniridia usually have acceptable tear functions and good lid appositions, making the Boston type 1 KPro a more suitable option.

All but one case improved their VA after the surgery, which is similar to other published studies (5, 6, 11). Nevertheless, 50% of the cases achieved ambulatory vision (VA > 0.025 decimal) (14) and functional success (BCVA ≤ 1.3 LogMAR) at the end of the follow-up period, with this value being the limit for legal blindness as defined by the WHO. These patients had vision good enough to see large objects at close range and the ability to move around in a familiar environment. These results are comparable with the 43.5% obtained by Shah et al. after 4.5 years of follow-up (1).

The probability of maintained VA over legal blindness limits in our study population was 57 and 46% at 1 and 5 years, respectively, to our knowledge, this is the first time a Kaplan–Meier survival analysis has been performed. In accordance with other authors, we attribute the limitation of achieving greater visual acuity in these patients to preexisting aniridia-related comorbidities such as foveal hypoplasia, nystagmus, and optic nerve hypoplasia (1, 2, 6). Moreover, the factors that tapered the VA in our cohort were retinal detachment (33%) with macular involvement and a high prevalence of preoperative glaucoma (100%) and its progression. Nearly all publication considered glaucoma as a major cause of visual loss that could have impacted the visual outcome results (1, 2, 5, 6, 11). Retinal detachment was also described by Akpek et al. as the cause of visual impairment in one of 16 cases and by Hassanaly in 31% of cases (2, 11). Our current surgical technique includes a planned pars plana vitrectomy (PPV) to reduce the risk of postoperative retinal detachment.

Retroprosthetic membrane formation (66%) was the most common complication in our study. Rudnisky et al. found a comparable rate (66.7%) in aniridia cases (15). This was significantly higher than the RPM formation in all Boston type 1 patients, including other categories (31.7%) (15). They were able to demonstrate that aniridia increased the hazard of RPM formation by 3.13 (95% confidence interval 1.1–8.89). Since 2013, the Centro de Oftalmología Barraquer has only been using titanium backplates, as they seem to be associated with less RPM formation than polymethyl methacrylate (PMMA) (16).

The frequency of complications and the high need for postoperative procedures in our study group add evidence that there might be a physiopathologic process in these patients that makes them unique compared with non-aniridic cases (1). This could be due to aniridia fibrosis syndrome (5). This suggests that aniridia should be considered a separate category in the stratification by the preoperative diagnoses of KPro patients rather than be included in the recurrent immunologic rejection category (7). Because of the high rate of complications, we recommend not to implant bilateral Boston type 1 KPro simultaneously.

A limitation of this study is its retrospective design and limited sample size due to the low incidence of the disease. A quality-of-life questionnaire could have added valuable data to the study. However, this study provides valuable information to the existing literature, especially related to the long follow-up time.

Based on our results, we conclude that the Boston type 1 KPro is a viable option for the treatment of AAK, with good long-term results both anatomically and functionally. Uncontrolled glaucoma is an important condition that affects functional results. Aniridia may be considered a separate category in Boston type 1 KPro stratification, but this needs to be validated by future studies.

## Data Availability Statement

The original contributions presented in the study are included in the article/supplementary material, further inquiries can be directed to the corresponding author/s.

## Ethics Statement

Ethical review and approval was not required for the study on human participants in accordance with the local legislation and institutional requirements. Written informed consent to participate in this study was provided by the participants' legal guardian/next of kin.

## Author Contributions

AD and ADF: research design, data acquisition, research execution, data analysis, interpretation, and manuscript preparation. GJ: data analysis, interpretation, and manuscript preparation. RB: data analysis and interpretation. JÁ: data analysis and interpretation. MP: research design, data analysis, and interpretation. All authors contributed to the article and approved the submitted version.

## Conflict of Interest

The authors declare that the research was conducted in the absence of any commercial or financial relationships that could be construed as a potential conflict of interest.

## Publisher's Note

All claims expressed in this article are solely those of the authors and do not necessarily represent those of their affiliated organizations, or those of the publisher, the editors and the reviewers. Any product that may be evaluated in this article, or claim that may be made by its manufacturer, is not guaranteed or endorsed by the publisher.
